# *In Vitro* Studies of Neuronal Networks and Synaptic Plasticity in Invertebrates and in Mammals Using Multielectrode Arrays

**DOI:** 10.1155/2015/196195

**Published:** 2015-03-17

**Authors:** Paolo Massobrio, Jacopo Tessadori, Michela Chiappalone, Mirella Ghirardi

**Affiliations:** ^1^Neuroengineering and Bio-Nano Technology Lab (NBT), Department of Informatics, Bioengineering, Robotics and System Engineering (DIBRIS), University of Genova, Via all'Opera Pia 13, 16145 Genova, Italy; ^2^Department of Neuroscience and Brain Technologies, Istituto Italiano di Tecnologia, Genova, Via Morego 30, 16163 Genova, Italy; ^3^“Rita Levi Montalcini” Department of Neuroscience, University of Torino, Corso Raffaello 30, 10125 Torino, Italy

## Abstract

Brain functions are strictly dependent on neural connections formed during development and modified during life. The cellular and molecular mechanisms underlying synaptogenesis and plastic changes involved in learning and memory have been analyzed in detail in simple animals such as invertebrates and in circuits of mammalian brains mainly by intracellular recordings of neuronal activity. In the last decades, the evolution of techniques such as microelectrode arrays (MEAs) that allow simultaneous, long-lasting, noninvasive, extracellular recordings from a large number of neurons has proven very useful to study long-term processes in neuronal networks *in vivo* and *in vitro*. In this work, we start off by briefly reviewing the microelectrode array technology and the optimization of the coupling between neurons and microtransducers to detect subthreshold synaptic signals. Then, we report MEA studies of circuit formation and activity in invertebrate models such as *Lymnaea*, *Aplysia*, and *Helix*. In the following sections, we analyze plasticity and connectivity in cultures of mammalian dissociated neurons, focusing on spontaneous activity and electrical stimulation. We conclude by discussing plasticity in closed-loop experiments.

## 1. Introduction

Communication among neurons is essential for higher brain functions, such as perception, memory, and movement. The mature nervous system is an intricate network in which neurons are extensively interconnected to each other. These connections are made up during embryonic and postnatal development and are modified during life by experience. As suggested by early experiments of Sperry [1–3], during circuit development axon-target recognition relies on chemical matching. Intercellular signals such as adhesion molecules, chemoattractants, and neurotrophic factors, remarkably well conserved during evolution, provide crucial directions to the developing nervous system [4–6]. In addition, electrical activity in the forming circuits also plays a critical role in shaping connectivity, as shown by the pioneering experiments of Hubel and Wiesel in the development of the visual cortex [7–11].

Activity-dependent fine-tuning of neuronal circuitry is not limited to early development and neural circuits are adaptable even in the mature individual [12]. As firstly proposed by Hebb in the 1940s, synaptic strength increases when the pre- and postsynaptic elements are synchronously active [13]. The existence of synapses whose efficiency is remarkably influenced by previous activity and that undergo long-term potentiation (LTP) has been discovered in rabbit hippocampus by Bliss and Lomo [14] and then long-term depression (LTD) has been observed in the cerebellar cortex [15].

Synaptic plasticity has long been implicated in cognitive processes such as learning and memory [16–18]. Interestingly, the same processes of plasticity described in simple nervous systems of invertebrates such as* Aplysia*,* Helix*,* Lymnaea*,* Helisoma*, and* Drosophila* seem to be conserved in mammals and many similar molecular mechanisms underlining both simple and complex forms of learning and memory [16, 17].

In the last decades, cellular and molecular aspects of synaptic plasticity have been analyzed in detail in circuits of few cells, while less information is currently available about dynamics of large populations of neurons during development and plastic modifications. Experimental analysis of connectivity with simultaneous multiple site recordings within functional neuronal networks is therefore a promising approach in neuroscience research.

Nowadays, microelectrodes arrays (MEAs) are the state of the art for studies on neuronal network dynamics. By means of those devices it is possible to characterize the neuronal dynamics of several biological preparations (i.e., from invertebrates [21, 22] to different cerebral mammalian areas such as cortex [23] and hippocampus [24]) by studying their development [25] and delivering electrical [26–28] or chemical stimulation [29–31] to induce synaptic plasticity at the network level [20]. Recently, improvements to the MEA technology have led to an increase in the number of recording sites [32–36]. In this way, the idea to have one neuron plated over the surface of each electrode (1 : 1 coupling) becomes achievable, thus implying the possibility of realizing neuronal networks with a precise and well identified topology. Signal recording systems (microtransducers) based on MEAs and field effect transistors (FETs) have been established as powerful tools for recording the electrical activity of networks of neurons cultured* in vitro* [30, 37–41]. Under this experimental condition, neurons are directly connected to the microtransducers by a neuroelectronic junction, and the neuronal electrical activity is noninvasively extracellularly recorded over long periods of time.

## 2. Microtransducer Array Technologies

### 2.1. Passive Microtransducers

MEAs are made of cell-sized electrodes (10–100 *μ*m diameter) placed onto a glass or on a printed circuit board substrate. The electrodes, typically made of gold, indium tin oxide (ITO), titanium nitride (TiN), or black platinum, are biocompatible, long-lasting, and preferably with a low impedance (less than 500 KΩ at 1 kHz) for low thermal noise. The MEA surface and electrode leads are coated with biocompatible insulators (e.g., polyamide or silicon nitride/oxide) which prevent short circuits with the electrolyte bath. These insulators, coated with adhesion molecules such as polylysine and laminin, allow and help neuronal coupling to the device surface. Finally, the low impedance of the electrodes and the choice of a correct voltage range for avoiding the generation of neurotoxic redox complexes allow the delivery of external stimuli.

The fabrication of MEAs is based on the thin-film technology [42]. MEAs can be grouped on the basis of the number of electrodes which define the array (from 60 to 10,000 electrodes), the electrode size (from 10 up to 30 *μ*m), and the interelectrode spacing (from 20 up to 500 *μ*m). Most of the results presented in this review are based on MEAs with 60 flat round electrodes made of titanium nitride. Tracks and contact pads are made of titanium or ITO, and the insulation material consists of silicon nitride (Si_3_N_4_). The electrodes are positioned in an 8 × 8 layout grid with missing corner electrodes. ITO contact pads and tracks are transparent to allow a perfect view of the specimen under the microscope. Finally, a glass ring is placed at the center of the array in order to contain the culture medium. In this way the cultures may survive for several weeks when placed in an incubator.

### 2.2. FET-Based Microtransducers

**Figure 1:**
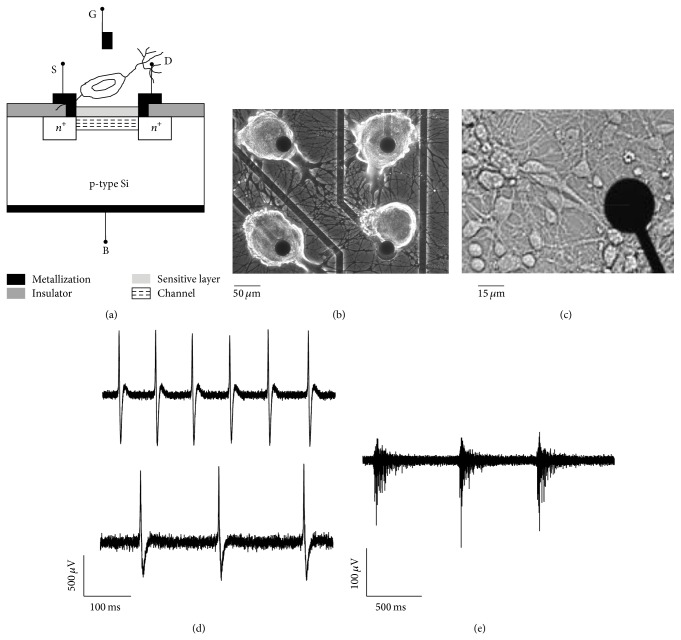
(a) Schematic representation of a field-effect transistor (FET). (b) Example of* Helix* neurons 24 hours after plating on MEA. Scale bar is 50 *μ*m. (c) Cortical neurons after 24 days* in vitro* coupled to a MEA. Scale bar is 15 *μ*m. (d) Extracellular action potentials recorded by a MEA relative to B2 (top) and C1 (bottom)* Helix* neurons. (e) Typical bursting activity of a cortical network recorded by a MEA.

A considerable contribution in the microtransducer field for electrophysiological neuronal activity recording was made by Fromherz and coworkers [37, 40, 43–46]. They demonstrated that open-gate FETs are able to detect the transient extracellular voltage beneath a single neuron attached, through its cell membrane, to the gate insulator of a FET. Briefly, a field-effect transistor consists of four terminals (Figure 1(a)): the source (S), the drain (D), the gate (G), and the bulk (B). The region between the source and drain defines the channel. The gate is separated from the channel by an insulator (SiO_2_ or Si_3_N_4_). The source and drain connections are degenerately doped* n*-type silicon, while the bulk of the transistor is* p*-type. If there is any kind of voltage applied to the gate, the source and drain are electrically disconnected. If a positive voltage is applied to the gate, positive charges in the gate will electrostatically repel the holes in the underlying* p*-type channel. If the applied bias is further increased, the positive charges on the gate will attract minority carriers (electrons) and a channel that will link the source and the drain together will take place, allowing the passage of current. Thus, one can modulate the drain-source current by applying a particular gate voltage. For the use of a FET as microtransducer to record electrophysiological activity, the metallic gate is removed. In this way, the insulator is directly exposed to the cell membrane and to the electrolytic solution. The activity of the neuron leads to ionic and displacement currents flowing through the attached membrane, resulting into an extracellular voltage drop along the narrow cleft between the membrane and the gate insulator. The change of the extracellular voltage induced by the neuronal activity gives rise to an electric field across the insulator which modulates the drain-to-source current of the FET; this current, transduced into a voltage, describes the extracellular recorded signal probed by the microtransducer. Compared to the first generation of FET-based systems [37, 46], nowadays low-noise level FET-based systems are available. The introduction of low-noise transistors [47] permits recording the electrophysiological activity also from mammalian neurons (cortical or hippocampal neurons) which exhibit a peak-to-peak amplitude smaller (~100 *μ*V) than the one originated by large invertebrate neurons (up to tens of millivolts). We can then conclude that, with respect to conventional MEAs, three main advantages can be achieved by using this kind of technology: (i) heavy parallelization*—*large numbers of microtransducers can be addressed by on-chip multiplexing architectures. In this way, it becomes feasible to design array of microtransducers with thousands of recording sites. This kind of devices can be exploited to study the emerging connectivity in large-scale neuronal networks; (ii) signal quality*—*the signal is conditioned right at the transducer by means of dedicated circuitry units; (iii) ease of handling and use—both devices and signals are robust.

### 2.3. Increasing the Coupling between Neurons and Microtransducers

In the last decades, several attempts have been made to improve the coupling between neurons and the surface of the microtransducers with the aim of increasing the amplitude of the recorded signals and achieving more stable adhesion conditions. In this sense, a significant result has been obtained by the use of carbon nanotubes (CNTs), considered a promising material for the assembly of nanodevices. Recent studies have suggested the great potential of high-density CNT coated surfaces as an interfacing material with neuronal systems; moreover, CNT surfaces act as an extremely efficient biocompatible substrate on which neurons adhere and proliferate [48–51]. Furthermore, the CNT-based neuroelectronic junction [52] plays a relevant role on the extracellular neuronal signal recording [53] and stimulation [54].

More recently, a great step towards the possibility to enhance the coupling between electrogenic cells and microtransducers has been made by Spira and coworkers who developed an innovative extracellular recording technique, based on mushroom-shaped protruding microelectrodes [55, 56]. This extracellular system allows* in-cell recording* not only of action potentials, but also of subthreshold synaptic inputs from individual neurons with a signal-to-noise ratio that matches that of conventional intracellular recordings.

## 3. Invertebrate Neurons and MEA Microchips

Invertebrate neurons have represented an important tool in the development of microtechnologies applied to neurosciences. Specifically, invertebrate neurons have a large cell body which facilitates the formation of high quality neuron/microtransducer interfaces; they are easily identifiably amid the ganglia and may grow in isolated culture as well as in reconstructed specific circuits. In addition, invertebrates neural circuitry is very simple when anatomically compared to mammals, but it exhibits many types of short and long-term plasticity that have been extensively studied at the behavioral, cellular, and molecular levels [17, 57]. Moreover, by exploiting the size of their somata and the amplitude of their action potentials, invertebrate neurons have also been used as testbed for innovative microtransducers. In 1991, a Retzius cell of the leech* Hirudo medicinalis* was successfully coupled for the first time to a transistor by Fromherz and colleagues [37, 58], and bioelectrical signals were elicited from neurons of the pond snail* Lymnaea stagnalis* grown on microchips and connected by gap junctions [44, 45, 59–61].

### 3.1. Chemical Connections and Plasticity in* Lymnaea* Neurons

A further important step has been the reconstruction on the silicon chip of a chemical synapse of* Lymnaea* that exhibited plastic properties [62]. In* Lymnaea*, the respiratory neuron VD4 (visceral dorsal 4) forms a cholinergic synapse with the neuron LPeD1 (left pedal dorsal 1) [63, 64] and this connection can be reconstituted in culture in a soma-soma configuration [64–68]. The VD4-LPeD1 synapse undergoes short-term plastic changes and intracellular tetanic stimulation of VD4 induces an enhancement of synaptic transmission in the postsynaptic neuron LPeD1 [69, 70]. Interestingly, similar potentiation was obtained when the presynaptic neuron VD4 was repeatedly stimulated by a chip capacitor and postsynaptic excitation in LPeD1 was recorded by a transistor, establishing for the first time a short-term plasticity in a pair of chemically connected identified cells cultured on the electronic chip [62]. This result provided evidence that interfacing semiconductor chips and neurons may represent a direct noninvasive method for short- and long-term studies of plasticity* in vitro*.

Simultaneous recordings from a large number of neurons of central pattern generator (CPG) networks that mediate fundamental* Lymnaea* behaviors including feeding and respiration [71–74] have been obtained from semi-intact preparations consisting of ganglia and sensory input interfaced to MEAs [75]. In this preparation the MEA was used to monitor learning-induced changes in the electrical responses of specific types of identified buccal feeding motoneurons [76–83]. Using a protocol for* in vitro* single-trial classical conditioning of* Lymnaea* feeding behavior [84], plastic changes were induced in the semi-intact preparation. Recordings with MEA technology have allowed detecting modifications in the spatiotemporal firing patterns of a large number of feeding neurons. This proved that the feeding CPG of the pond snail is associated with another oscillating neuronal population [85] characterized by activity alternating with quiescence during which the CPG is refractory to activation by food-associated stimuli. Similar network refractory periods have previously been observed in the rhythmic activity networks of spinal interneurons from locomotor CPG regions in culture as well as in the spinal cord of embryonic rat [86, 87] and chick [88]. In* Lymnaea* circuits interfaced with MEA, the dynamic of network refractory periods of CPG was modulated by dopamine [85], a neurotransmitter known to regulate feeding behavior and reward [89].

### 3.2. Multisite Detection of Dopamine Release from* Lymnaea* Neurons

Recently multifunctional MEAs were developed for neuroelectrical and neurochemical recordings* in vivo* [90, 91] and* in vitro* that allowed the detection of neurotransmitters such as dopamine released from neurons of acute hippocampal slices [92] and from PC12 cell lines [93]. Neurotransmitters released from presynaptic terminals regulate neuronal communications and it is well known that alteration in electrical activity and in level of neurotransmitters underlies several disorders such as Parkinson's disease, schizophrenia, and major depression [94, 95]. Multimodal probes for simultaneous activity and chemical detection appear suitable for analyzing dynamics of activity correlated to neurochemical release in complex circuits and for investigating the effects of drugs employed in treatments of neurological diseases.

More details on the specific sites of neurotransmitter release from a neuron have been acquired with a planar microelectrode array that has been fabricated and characterized by Patel et al. [96] for simultaneous multisite detection of dopamine release. Electrically evoked dopamine release was observed from freshly isolated dopaminergic RPeD1 neurons of* Lymnaea stagnalis*. These large neurons were plated on MEAs containing electrodes spatially arranged to allow the simultaneous recordings from various structural regions of an isolated cell. Evoked recordings of dopamine release induced by tetanic stimulation were obtained simultaneously from distinct locations such as the soma, the axon, and the terminals. Interestingly, repeated recordings at various time-points showed that the release of neurotransmitter varied over the structural regions of the* Lymnaea* neuron during the reorganization of the cell following isolation from the ganglia. MEA recordings of simultaneous spatiotemporal responses of dopamine released by action potential activation from cultured large neurons could be used to study the changes in neurotransmitter release in specific regions of the neurons during formation of synapses and neuronal network activity.

### 3.3. “In-Cell” Recording in* Aplysia* Neurons

Many extracellular electrodes such as noninvasive extracellular MEAs for* in vitro* recordings can reliably measure action potentials but subthreshold potentials such as synaptic potentials remain undetectable. A large number of events in synaptic plasticity are associated with changes in amplitude of synaptic potentials that very often do not reach the threshold required for spike firing [16]. In the last decade, many attempts have been performed to improve the electrical coupling between cultured cells and planar MEAs and to reduce the junctional membrane resistance. A novel neuron-electrode configuration (“in cell” recording) developed by the group of Spira [55, 56, 97–100] allowed recording action potentials as well as synaptic potentials with MEAs in* Aplysia* neurons: it consists of an array of noninvasive gold, mushroom-shaped microelectrodes that permits simultaneous, multisite, long-term recordings of action potentials and subthreshold potentials with quality and signal-to-noise ratio comparable to that obtained with intracellular sharp glass microelectrodes or patch electrodes.

Neurons from the buccal and abdominal ganglia of* Aplysia californica* were isolated with their initial axon and maintained in culture [101, 102]. Plated* Aplysia* neurons survive in culture for over a month, extending neurites and forming chemical and electrical synapses [103]. Isolated* Aplysia* neurons were manually plated directly on top of the microelectrodes that protrude from the glass substrate with three-dimensional mushroom geometry and dimensions that mimic dendritic spines in their shape and sizes. A peptide that induces phagocytotic activity was covalently linked to the microelectrodes in order to generate efficient contact between them and the neurons. Electron microscopy showed that the neuron-microelectrode has a reduced cleft width with increased contact area [56] and within a few minutes of contact there was a restructuring of the actin cytoskeleton to form an actin ring around the stalk of the microelectrode that became a stable cytoskeleton structure and was maintained for several days as shown by live confocal microscopy [99]. Ultrastructural observations revealed that other cell types such as CHO, embryonic fibroblast cells NIH/3T3, rat myocardium cells H9C2, and rat adrenal medulla PC12 cell lines engulf the head and stalk of the gold spines [56]. The gold-spine matrix influences the growth of neurons but they maintain typical electrophysiological properties and form functioning synapses. These electrodes represent an improved substrate for the assembly of neuroelectronic devices well suited for the study of neuronal plasticity as they allow the detection of subthreshold potentials in* Aplysia* neurons.

### 3.4. *Helix* Circuits: Morphological and Electrical Development

Signals from single or paired invertebrate neurons have been recorded with MEA devices for many years but the first long-lasting study on invertebrate neuronal networks in culture was performed in 2013 by Massobrio and colleagues [19]. They characterized the dynamics and connectivity of networks made up of neurons of the land snail* Helix aspersa* coupled to MEAs during their development for several days. Previous experiments showed that C1, C3, and B2 neurons from buccal and cerebral ganglia of* Helix* form microcircuits* in vitro* [22, 104–107] and the serotonergic connection C1-B2 involved in the regulation of feeding behaviors of* Helix* snails reconstructed in culture undergoes plastic changes [105]. The large size of* Helix* neurons (soma diameter up to 100–150 *μ*m; see Figure 1(b)) compared to mammalian neurons (see Figure 1(b)) permits a 1 : 1 coupling between neurons and microelectrodes, facilitating the study of the relationships between the electrophysiological activity of individual neurons in a network and their development of neurite outgrowth and connections in the long term.

*Helix* neurons start to develop synaptic connections a few hours after plating, and their development is faster than cortical cultures from mammals [25].  Figure 1 shows the growth of* Helix* neurons that have been plated on top of the microelectrodes 24 hours earlier (1B) and of cortical neurons after 24 days in culture (1C).

**Figure 2:**
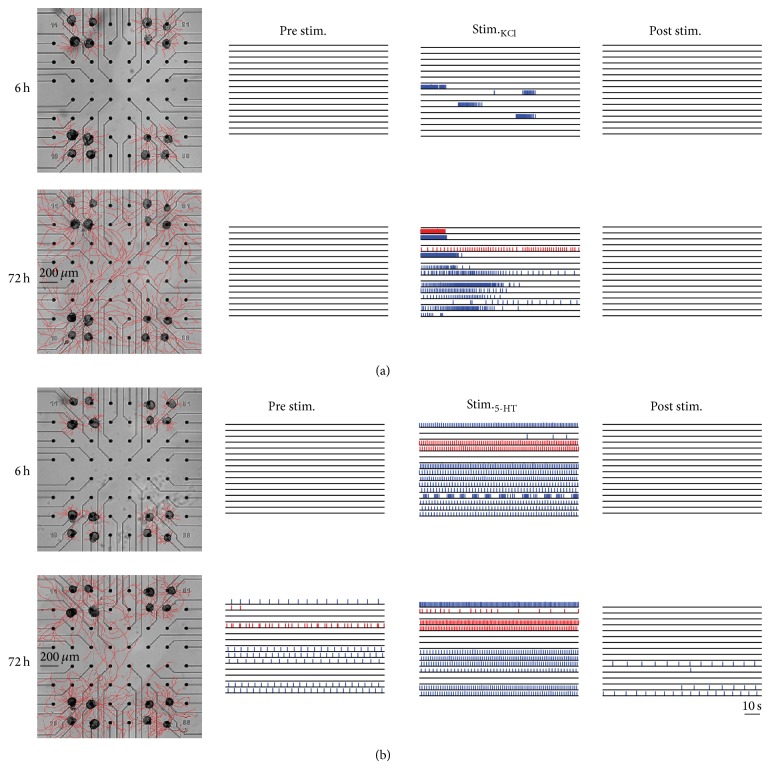
Example of* Helix* cultures treated with KCl (a) or 5-HT (b) applications. Each block represents a time-point of recordings (i.e., 6, 72 hours after plating). In the first column, the development of the neurite arborizations is indicated (scale bar is 200 *μ*m). The three columns of raster plots show one minute of electrophysiological activity just before, during, and after the KCl or 5-HT treatment. Red and blue lines indicate recordings from C1 and B2 neurons, respectively. Scale bar is 10 s. Modified from [19].

A peculiarity of these invertebrate neuronal networks is the absence of spontaneous activity:* Helix* neurons are generally silent on MEAs [19, 22, 108, 109], and spontaneous firing was observed only occasionally [22].  Figure 1(d) shows two examples of extracellularly recorded signals coming from stimulated B2 (top) and C1 (bottom) neurons. Typically, the signals from these neurons display amplitudes greater than 500 *μ*V, differently from mammalian spikes that, although spontaneously active, seldom are greater than 200 *μ*V.  Figure 1(e) shows a typical cortical bursting sequence. Therefore, to study* Helix* networks, neuronal activity was triggered by means of chemical treatments that induce general depolarization of the cell membrane potential (potassium chloride, KCl, Figure 2(a)) or that selectively depolarize B2 neurons (serotonin, 5-HT, Figure 2(b)). Thus, the neuritic outgrowth and the induced activity were followed during development of the circuits for several days, as shown in Figure 2. The first-order statistics were used to characterize neuronal dynamics such as interspike interval (ISI) and firing rate. The cross-correlation function [110] was employed to estimate the functional connections established among neurons of the network in order to reconstruct the topological connections and monitor them during development. The simplicity of these neuronal circuits allows the achievement of a good matching between morphological and functional links on MEA. It has been found that both chemical stimulations were efficacious in triggering firing activity in* Helix* circuits, but a long-lasting change in activity occurred only with 5-HT treatment, as presented in Figure 2. For as long as the network evolved, an increase with time of functional connections was detected. Moreover, the analysis of spiking activity as well as their functional linked latencies showed that networks treated with 5-HT displayed a dynamic modulated mostly by chemical synapses, while a predominance of electrical connections occurred in KCl-triggered networks.

The preferential formation and strength of chemical or electrical synapses during circuit development are critically regulated by several factors including neuromodulators such as serotonin and dopamine, as recently reviewed by Pereda [111]. The prevalence of chemical connections in 5-HT-treated circuits may underline the long-term maintenance of spontaneous activity. Serotonin applications trigger activity through the depolarization of B2 neurons and the increased excitability of C1 neurons, and continuous release of 5-HT from the firing neurons may maintain it, as previously reported [112–114]. In addition, it has been demonstrated that 5-HT selectively prevents the formation of electrical synapses while allowing chemical synaptogenesis between identified* Helisoma trivolvis* neurons [115, 116], maybe due to a negative modulation of neurite elongation [117–121] and a direct action on gap junctions [122–124], also observed in neocortical circuits [125]. In mammals, 5-HT has been implicated in shaping neuronal connectivity by decreasing neurite branching in rat cortical neurons during development [126] and impairing neurite density in mouse organotypic slice cultures [127]. The increase in neurite density induced by KCl treatments may contribute to enhancing cell-cell contact, thus promoting a higher coupling coefficient among cells [118]. Since gap junctions likely play a fundamental role in determining network synchronization [128, 129], signals may reverberate among neurons until they return to a silent state by switching off the circuit.

Following repeated stimulations, similar dynamics during the development of these invertebrate circuits were observed: network activity soon reached values of firing rates and ISIs which remained almost unchanged during the development despite connectivity maturation. This behavior is very distinctive, especially if compared with studies regarding the development of* in vitro* cortical neurons from rat embryos [25]. Indeed cortical assemblies were found to change their electrophysiological patterns as a function of network maturation, probably associated with a much higher synapse density [130, 131].

## 4. Plasticity and Connectivity in Mammals

### 4.1. Mammalian Neuronal Assemblies Display Spontaneous Electrophysiological Activity

Cultured neurons extracted from both embryonic and postnatal rodents are spontaneously active. This activity, originating from the interactions of neuronal assemblies, is a peculiar feature of the mammalian nervous system and it can be found at different levels of investigation: in the cerebral cortex, it takes the shape of oscillatory patterns which span over different rhythms [132]; in reduced* in vitro* models, like dissociated cortical cultures, spontaneous activity is mainly characterized by a mixture of spikes and bursts lasting from a few to hundreds of milliseconds [38]. Such dynamic evolves, since it changes as a function of the degree of development of the cultures [133]: during the first stages of development, dissociated cortical assemblies display mainly irregular and asynchronous spiking activity; from the second week* in vitro*, spikes tend to cluster into bursts, a signature feature that persists throughout the time in culture, thus representing the mature state of the network. Those bursts can be found in both hippocampal [24] and cortical cultures [134] and are similar to activity patterns of* in vivo* systems deprived of afferent stimuli or with pathologies like epilepsy [135]. On the other hand, Eytan and Marom [136] noted similarities between the dynamics of these* in vitro* “synchronized bursting events” and population responses recorded* in vivo* while animals are engaged in sensory, motor, or “internal” cognitive tasks.

The presence of this “cumbersome” ongoing activity, exhibiting a high degree of variability, makes it difficult to interact with these cortical ensembles. However, several attempts to modulate these irregular dynamics by means of appropriate electrical and/or chemical stimulations can be found in the literature. In general, low-frequency, uniform, sustained electrical stimulation locks the phase of periodic bursts to the applied stimuli [137]. Higher rates of stimulation induce a transition from synchronized bursting activity into a sparse spiking behavior, more similar to* in vivo* awake cortical dynamics [27]. Conversely, an electrical stimulation pattern tailored on the network endogenous activity is able to efficiently induce modifications in the network synchronization and, in particular, it affects the network bursting properties, by increasing both firing and bursting rate [138]. Moreover, after this kind of spontaneous activity-tailored stimulation, the strongest connections respond by further increasing their strength relative to other connections within the network. This mechanism likely preserves connections that are more informative and relevant to the overall network activity.

### 4.2. Searching for Plasticity with MEAs: A History 20 Years Long

The history of MEA studies demonstrating functional plasticity in cultured mammalian networks began in the 1990s with the pioneering work of Maeda and coworkers who reported that tetanic stimulation through one or more electrodes was able to induce plasticity [139]. In their work, they found that the probability of evoking bursts by test pulses, as well as a change in the rate of spontaneous bursting, was modified after the delivery of a strong tetanic stimulation. Less than one year later, Jimbo and coworkers observed similar results with a milder tetanic stimulation [140]. Following those experiments, numerous other attempts have been performed by several MEA labs worldwide aimed at finding whether peculiar features of the electrical stimulation (e.g., frequency, number of stimulated electrodes, and amplitude of the stimuli) were able to induce synaptic changes in the dynamics of the networks.  Table 1 summarizes the most significant plasticity studies.

However, despite the different attempts pursued during these years, a clear answer to the question of whether neuronal cultures can learn thanks to plasticity phenomena is still controversial [147], mainly because of two reasons: the first is the difficulty of designing a network stimulation protocol (partly linked to the nonstationary behavior of the dissociated cultures) capable of reliably inducing changes, as we will report in the next sections. The second reason lies in the lack of an “electrophysiological endpoint” that can be easily correlated to plasticity in dissociated networks. Regarding the latter, most scientists supported the hypothesis of Marder and Buonomano [148]. They proposed that not only changes in the synaptic potentials but also changes in the firing patterns of neurons should be taken into account in long-term forms of plasticity. Moreover, the neuronal input/output function of the entire network must be studied and characterized to better understand the computational effects of plasticity on a long-term perspective [148]. According to this approach, MEA could become the gold-standard tool to define long-term network plasticity (LTNP) experiments [20]. Similarly, in 2006 Potter's lab coined the expression functional plasticity (FP) to indicate “those changes in stimulus-response relationships or in spontaneous patterns that are experimentally induced by electrical stimulation and lasting at least on the order of one hour” [145]. Phenomena like LTP and LTD fall within the boundary of this definition (and can thus be considered examples of functional plasticity), whereas short-lasting changes such as paired pulse facilitation and depression do not (their duration is much shorter than the one-hour limit).

In 2006, Potter's group published an interesting paper in Journal of Negative Results in Biomedicine. The authors applied different protocols to induce plasticity to a large set of cortical cultures coupled to MEAs [145]. The conclusions of their work are straightforward: bursting suppression obtained through distributed electrical stimulation is a prerequisite for inducing plasticity [27]. Their protocol consisted in the random selection of electrodes in the pool of those evoking electrical responses, followed by stimuli delivery in cyclic order, with an interstimulus interval of 20 ms. This resulted in a complete, but reversible, cessation of spontaneous bursting. Only after this was achieved, the application of a tetanic stimulation resulted in plasticity in their experiments.

The works of other groups determined the fact that complete bursting suppression is not a strict prerequisite and, through appropriate experimental precautions, different tetanic stimulation protocols resulted in significant connectivity changes even in the presence of spontaneous bursting. Several of those papers are discussed in more detail in the following chapters.

### 4.3. Stable Recordings Are Necessary to Induce Plasticity

**Figure 3:**
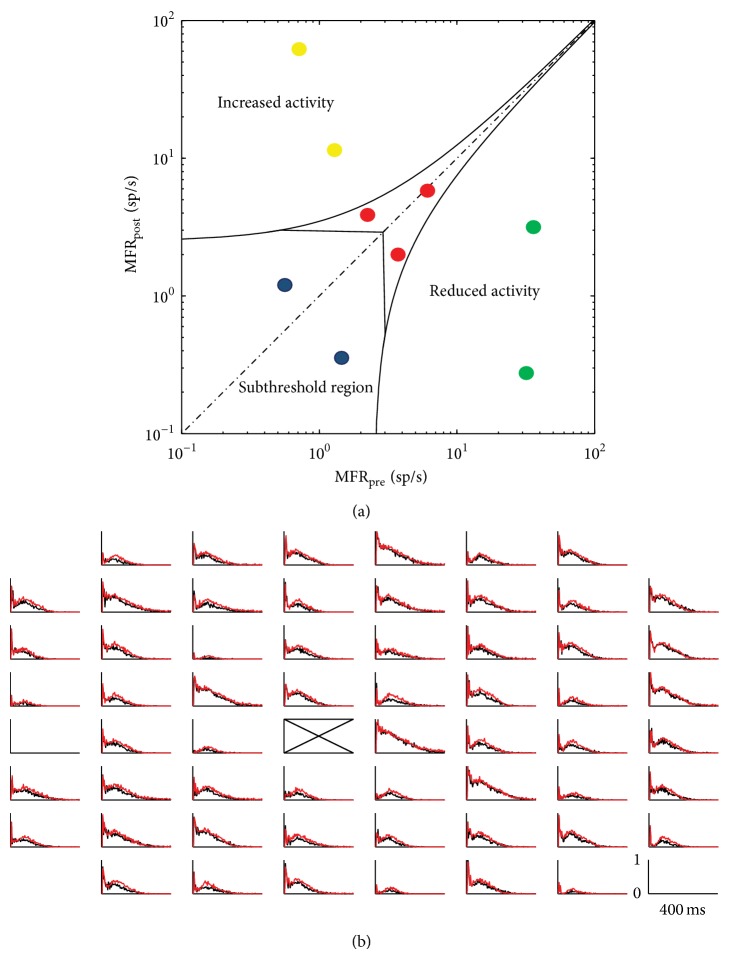
Initial conditions necessary to induce plasticity in cortical cultures. (a) Bilogarithmic plot of the mean spontaneous firing rates of cortical neurons. Spontaneous activity of each network was measured at the beginning of the experiment (MFR_pre_) and after the first test stimulus session (MFR_post_). Curved lines denote confidence intervals (see [20]). The used colors have the following meanings: red circles represent cultures that verify the MFR stability conditions and are potentially able to show plastic behavior. Blue circles indicate culture with a firing rate too low, while yellow and green circles represent unstable cultures which increase (yellow) or decrease (green) their firing rate. (b) PSTH map. This example shows the effects on the network during the stimulation from the site indicated by the cross. The two superimposed profiles of the PSTH represent the responses of the network to two different sessions of stimulation. Bin size = 4 ms; *x*-axis [0,400] ms; *y*-axis scale [0,1] is the probability to evoke spikes.

Stability and responsiveness of culture batches are the first and most important conditions to be assessed to induce plasticity. As stated in the previous sections, both cortical and hippocampal assemblies display a mixture of spiking and bursting activity, with a high degree of variability (e.g., percentage of random spiking activity and frequency of the bursting activity) from culture to culture. For this reason, Chiappalone and coworkers developed a procedure that evaluates the level of nonstationarity of the spontaneous and stimulus-evoked activity of a neuronal network [20]. The application of this criterion allows discarding of cultures displaying large variations of dynamics which can obscure changes in the synaptic efficacy induced by plasticity protocols. Cultures are selected on the basis of their initial spontaneous activity and on their ability to respond to low-frequency stimulation. In particular, two conditions regarding the spontaneous activity and two conditions regarding the stimulus-evoked activity should be met. Firstly, the initial value of firing rate should be above a defined threshold (set at 3 in [20]), and secondly the firing rate should remain stable between the phases of spontaneous activity before the plasticity protocol delivery. As for stimulus-evoked activity, a stimulated electrode is not considered for future stimulations if it is not able to evoke a global response in at least 50% of the recording electrodes. Finally, the network response to low-frequency (i.e., 0.2 Hz) “test” stimuli has to be stable; that is, the variation of the evoked response, evaluated by means of the peristimulus time histogram (PSTH), has to be below a defined threshold.  Figure 3(a) shows a bilogarithmic plot of the mean spontaneous firing rates. Spontaneous activity of each network was measured at the beginning of the experiment (MFR_pre_) and after the first test stimulus session (MFR_post_). Curved black lines define the confidence intervals (see [20]). Cultures labeled with red circles verify the MFR stability conditions and are potentially able to show plastic behavior. On the other hand, blue circles indicate cultures with a low firing rate, while yellow and green circles represent unstable cultures exhibiting spontaneous increase (yellow) or decrease (green) of their firing rate.  Figure 3(b) shows an example of a PSTH map. The two superimposed profiles of the PSTH represent the probability of response (*y*-axis: [0,1]) of the network to two different sessions of stimulation (black and red) in a time window of 400 ms (*x*-axis: [0, 400 ms]). We can observe that (i) all the electrodes are responsive and (ii) the two traces are practically superimposed, thus indicating stability of the preparation.

### 4.4. Two Families of Plasticity Protocols: Slow versus Fast Electrical Stimulation

The protocols currently used to induce plasticity both* in vitro* [14, 149] and* in vivo* [150, 151] consist of a massively synchronous, high-frequency stimulation (40–100 Hz) named tetanic stimulation. Although these patterns of stimulation are extremely efficient, they have the drawback to rarely occur in nature [152]. The pioneering works which made use of tetanic stimulation also in cortical cultures coupled to MEAs were carried out in the groups of Maeda [139] and Jimbo [140]. Maeda and coworkers found that tetanic stimulation delivered by means of one or several electrodes induced plasticity [139]. They observed a change in the probability of evoking bursts by test pulses, as well as a change in the bursting rate of the spontaneous activity. Jimbo et al. observed similar results with weaker* tetani* and used voltage clamp to observe inward currents associated with evoked bursts [140]. The intriguing result was that, after tetanization, the onset latencies of these currents resulted were shorter. The following year, Jimbo et al. reported that tetanizing a single electrode resulted in changes in the responses to test pulses delivered from other electrodes [141].

Following this approach, Chiappalone and coworkers designed a more physiological experimental protocol derived from the combination of previous works on associative stimulation for LTD and LTD induction [153, 154] and on the results of the Japanese groups [139, 140]. The applied tetanus was characterized by a sequence of short bursts at 20 Hz every 5 s coupled to a stimulation below 1 Hz for a limited period of time (less than 2 minutes) [20]. The obtained results, based on different pairing of the tetanus with the weak train of stimuli, demonstrated that reliable potentiation was obtained by using more physiological stimulation and without drastically changing the natural spontaneous dynamics of the neuronal system. Moreover the obtained changes were long-lasting since observation up to twenty-four hours after tetanization. Those findings do not contradict the findings of Jimbo et al. [140, 141], according to whom the tetanic stimulation was, by itself, able to induce plasticity in the network. The main result is that the single tetanus is less reliable and it involves a smaller fraction of (effective) connections but its efficacy can be greatly increased when paired with a weak stimulation [20]. A similar work was performed, in 2010, by Le Feber and coworkers [146]. They applied a low-frequency stimulation protocol (i.e., biphasic current pulses at a frequency of 0.2–0.33 Hz) to cortical cultures coupled to MEAs. Their analysis investigated possible modifications induced by stimulation on the network functional connectivity. Their main findings showed that stimulation is effective in inducing changes for as long as it triggers network bursts, with very little correlation with the actual stimulation frequency used. Furthermore, stimulation changed network activity patterns from the spontaneously observed ones: stimulation-triggered network bursts originate at other points than spontaneously occurring bursts and, therefore, spreading of activity involves different pathways.

The approach followed by the groups led by Torre [144] and Potter [155, 156] is completely different and based on more “classical” protocols for inducing plasticity* in vitro* [14, 157]: in these works, tetanization consists of multisite bursts of stimuli at 250 Hz or of continuous trains of electrical pulses at 20 Hz for 15 min. By using such massive, nonphysiological stimulation, the first study [144] reported increase in evoked firing at specific sites, without quantification of duration and amount of the changes. Madhavan et al. [156] reported an increase in global spontaneous activity, without specifically analyzing the evoked response.

### 4.5. Limitations and Perspectives on Plasticity in Dissociated Mammalian Cultures

Inducing plasticity by extracellular electrical stimulation in dissociated mammalian cultures has not been as straightforward as for brain slices [158]. The possible reason behind this could be related to the nonstationary behavior of the dissociated cultures (cf.  Section 4.2). Specifically, since much of the activity in cultures is concentrated in bursts (possibly caused by lack of critical neuromodulatory input during development [159]), these dynamics could quickly cancel the effect of plasticity [160]. Moreover synapses are already saturated in culture due to the high density of the established connections.

**Figure 4:**
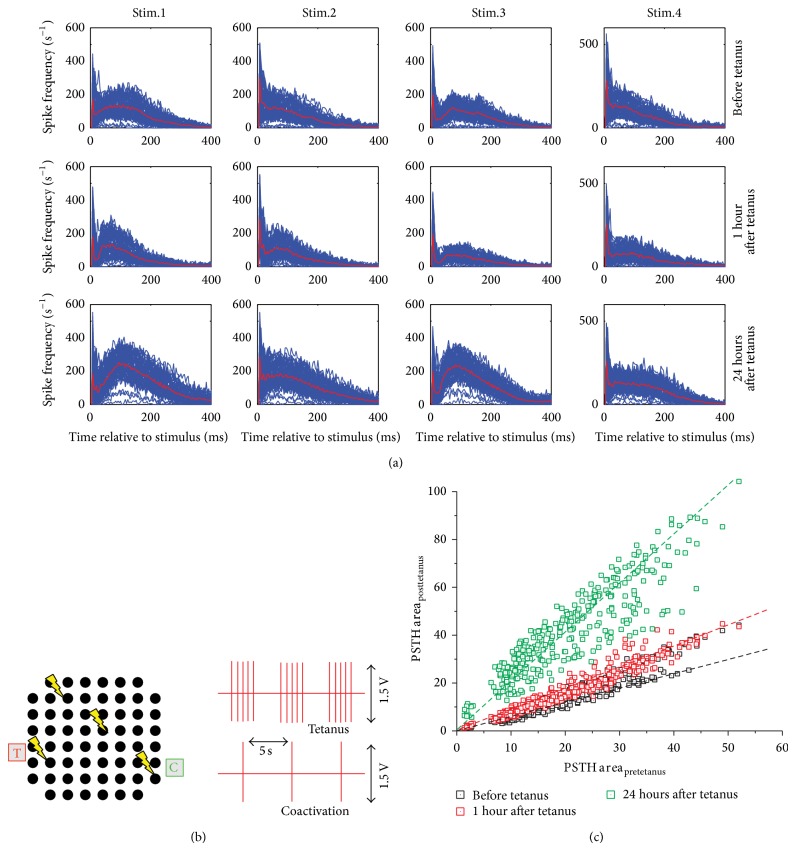
(a) Superimposition of the PSTH curves (each per electrode). The thick red line indicates the average shape. Each row indicates a peculiar phase of the protocol: before the delivery of the tetanus (first row) and 1 hour (second row) and 24 hours (third row) after delivery of the tetanus. Each column indicates a different stimulation site. (b) Sketch of the tetanic protocol described in [20]. Each black dot represents a microelectrode. The yellow arrows indicate where the stimuli are delivered. The tetanic protocol consists of a high frequency tetanic stimulation coupled to a low-frequency (in-phase) stimulation. (c) Scatter plot of the PSTH area for each active electrode. Each point represents an active channel with a certain PSTH area during three phases of the protocol: before the delivery of the tetanus (black squares) and 1 hour (red squares) and 24 hours (green squares) after delivery of the tetanus. The dashed lines represent the linear fitting of the three scatter plots.

However, this last motivation could also justify why it has been possible to induce plasticity, as demonstrated by the recent findings reported in this review: assuming a random probability to establish connections and that the chance of forming monosynaptic connections with nearby neurons is quite low [141], there would be a large number of recurrent polysynaptic pathways, as in the intact brain. The firing of a cultured neuron hyperinnervating others can be considered to be analogous to the* in vivo* situation of synchronous firing of a group of neurons with common targets [149]. For this reason, forms of heterosynaptic plasticity are more likely to occur in dense cultures than homosynaptic ones. For the same reasons, coupled stimulation (e.g., Chiappalone's protocol) mimics, at the cell assembly level, the heterosynaptic pairing as reported for the hippocampus [154], leading to a LTNP, in the form of long-lasting plasticity (i.e., L-LTP) which can be maintained for hours or even days. Figures 4(a) and 4(c) show an example of LTNP. In Figure 4(a) the PSTH shapes of signals from all the electrodes (blue lines) and their average (red thick line) trend are reported before the tetanus delivery (first row) and 1 (middle row) and 24 hours (bottom row) after the tetanus delivery. The tetanic protocol is sketched in panel (b). Qualitatively, a change in the shape of the response can be appreciated. To quantify such results, Figure 4(c) plots the area under the PSTH curves before tetanus delivery (black squares) and 1 (red squares) and 24 hours (green squares) after the tetanus delivery. A clear potentiation of the network can be observed and quantified by the slopes of the linear fittings (dashed lines). As reported in [149], synaptic plasticity at the network level provides a distributed mechanism to convert and store temporal information into spatially distributed patterns of synaptic modification.

## 5. Plasticity in Closed-Loop Experiments

Closed-loop experiments on* in vitro* neural networks have been introduced to investigate whether such preparations could perform a learning task without the need for a separate rewarding entity: in 2001, the group of Marom [143] implemented a relatively simple activity-dependent stimulation protocol that would cease once the firing of the network met a predefined threshold: they developed an activity-dependent adaptive stimulation protocol, aimed at training a culture to produce a predefined response upon stimulation. In this way, networks could be taught to respond in specific ways to test pulses, by repeatedly stimulating them until the desired response was obtained. This approach was based on general learning theories (stimulus regulation principle). In their experiments, the reward consisted in a reduction of the driving stimulus, precluding the acquisition of any new stimulus-response associations.

Similar experiments have been more recently conducted by the groups of Le Feber [146] and Kazantsev [161], in 2010 and 2013, respectively. In more detail, an electrode in a standard, 60-electrode MEA layout was used to deliver low-frequency (≤1 Hz) stimulation pulses. At the same time, the strength of functional connectivity between any pair of electrodes is estimated in real time, with stimulation protocols being interrupted when the functional connectivity between the stimulating electrode and a different arbitrarily chosen electrode in the MEA showed a significant increase. The results of these experimental campaigns proved that indeed closed-loop stimulation may be used to induce specific changes in network connectivity with no or very little* a priori* knowledge of the network structure.

Along the same line, Potter's group induced random changes in synaptic connections by stimulating in quick succession from several different electrodes, in order to induce a mix of potentiation and depression [162]. A stabilization pattern also consisted in stimulation from different electrodes, but interpulse intervals were chosen in order not to trigger spike timing dependent plasticity (STDP). In those experiments, the activity of the network resulted in the movement of an “animat” (i.e., an artificial animal): each time the behavior of the animat approached the intended one, the stabilization pattern was delivered; conversely, the network was stimulated with the training pattern to provoke a change of the animat behavior. The main result of that work was that the same patterns used for training did not cause any significant plasticity if delivered in open loop. However, this was not the only example of hybrid neural-artificial systems controlling a robot in closed loop. Several neurorobotic systems have been introduced and presented in the literature between 2007 and 2012 [163–166]. Differently from Potter's, those systems did rely on* a priori* knowledge of connectivity within the neural networks to successfully drive the robot past obstacles. These systems implement a closed-loop system that takes inspiration from a typical physiological sensory-motor loop: the sensors of the robot gather information about the surrounding obstacles, which is coded as a sequence of electrical stimuli delivered to the neural networks. The responses of the network are in turn decoded and used to safely drive the robot around its environment. These systems are generally intended as platforms for the study of specific aspects of neural network mechanisms, such as coding and decoding, or to test novel learning paradigms.

Identification of input-output relationships even for single neurons has proven intractable for a long time, given the timescales and nonlinearities involved. A step forward in this direction has been taken in 2011, when Marom's group introduced for the first time the concept of “neuronal response clamp” [167]. The idea is that of taking advantage of a proportional-integral-derivative (PID) controller to modulate the amplitude of a fixed rate electrical stimulation in order to maintain a stable response ratio at a given neuron. In this way, it is possible to estimate the threshold of the neuron, a high-level, functionally relevant variable. Thanks to the closed-loop neuronal clamp, in 2012 the same group investigated the interplay between network burst and single-neuron threshold [168]. They found the two phenomena to be deeply intertwined: the size of bursts results correlated with threshold values at the time of inset, while the dynamics of threshold recovery govern both spontaneous bursting and response to electrical stimulation. The effect of closed-loop stimulation on plasticity is presented in a more recent work by the same group [169]. They concluded that, in open-loop conditions, networks will respond to electrical stimulation whenever they have built up enough resources to do so; on the other hand, in a closed loop, networks are forced to keep up with stimulation rate: this imposes the recruitment of resources which are generally left unused. In turn, this leads to enhanced changes in connectivity compared to those observed in open-loop experiments.

## 6. Concluding Remarks

Simultaneous multisite long-lasting recordings with MEAs have opened new perspectives in the studies of formation and dynamics of complex neural networks allowing detailed investigations at the level of single cells and at population scales both* in vitro* and* in vivo*. In the last decades, technical improvements have increased spatial resolution of MEA recordings [36] and recently the detection of subthreshold signals such as synaptic potentials has been demonstrated in invertebrate neurons [97]. Future efforts will be focused on the development of strategies to record synaptic signals in mammalian neurons too, as well as to create more realistic connectivity, like 3D structures [170] or the presence of heterogeneous neuronal populations [171]. Studies of connectivity and synaptic plasticity in dissociated mammalian cultures will help to understand complex dynamics underlying both physiological behaviors and pathological alterations [172] associated with changes in firing patterns of large populations of neurons as it happens in epileptic disorders.

As reviewed in this work, the behavior of dissociated cortical networks can be shaped by the delivery of* ad hoc* stimulation patterns suggesting that this reduced* in vitro* experimental model is capable of learning or adapting to the timing of the stimuli. These conclusions have been extended in 2010 by Buonamano and coworkers that found that neuronal dynamics in a complex circuit can be modified through experience and that the temporal structure of such expressed dynamics reflects the temporal interval used during training [173].

Finally, the possibility to interface neuronal networks with MEAs allows the realization of neurorobotic frameworks. Such hybrid platforms are a valid tool for the study of mechanisms of neural coding and the computational and adaptive properties of neuronal assemblies [174]. In a long-term vision, these systems could be used to better understand neural pathologies, to design neural prosthetics, and to create different types of hybrid intelligence.

## Figures and Tables

**Table 1:** Summary of the plasticity protocols used in mammalian neuronal cultures.

Reference	Year	Plasticity protocol
Maeda et al. [139]	1998	20 bursts at 0.2 Hz, each with 11 pulses at an intraburst frequency of 20 Hz delivered from 5 electrodes

Jimbo et al. [140]	1998	11 bursts at 0.2 Hz, each with 11 pulses at an intraburst frequency of 20 Hz

Jimbo et al. [141]	1999	10 bursts at 0.2 Hz, each with 11 pulses at an intraburst frequency of 20 Hz

Tateno and Jimbo [142]	1999	10 bursts at 0.2 Hz, each with 11 pulses at an intraburst frequency of 20 Hz, also delivered from a pair of electrodes

Shahaf and Marom [143]	2001	Adaptive stimulation between a pair of electrodes at 1–3 s intervals repeated until the desired response is achieved (or for 10 min max)

Ruaro et al. [144]	2005	Bursts of 100 pulses delivered at the frequency of 250 Hz from 15 electrodes recreating an “L-shape”

Wagenaar et al. [25, 145]	2006	150 trains of 20 pulse pairs. Bursts suppressed by means of a high frequency (50 Hz) distributed stimulation (not during tetanus delivery)

Chiappalone et al. [20]	2008	Jimbo protocol with additional trains of pulses at 0.2 Hz falling in the middle of the tetanic burst

Le Feber et al. [146]	2010	Slow electrical stimulation (0.2–0.33 Hz)
